# Adipose Tissue-Derived Stem Cells Alleviate Cold Allodynia in a Rat Spinal Nerve Ligation Model of Neuropathic Pain

**DOI:** 10.1155/2020/8845262

**Published:** 2020-10-12

**Authors:** Hyeon Seok Jwa, Yong Ho Kim, Jaehee Lee, Seung-Keun Back, Chul-Kyu Park

**Affiliations:** ^1^Department of Health Sciences and Technology, GAIHST, Gachon University, Incheon 21999, Republic of Korea; ^2^Gachon Pain Center and Department of Physiology, Gachon University College of Medicine, Incheon 21999, Republic of Korea; ^3^Department of Physiology, College of Medicine, Korea University, Seoul 02841, Republic of Korea; ^4^Department of Biomedical Laboratory Science, College of Medical Science, Konyang University, Daejeon 158, Republic of Korea

## Abstract

Neuropathic pain caused by lesions or nervous system dysfunction is a neuroimmune disease with limited therapeutic options. Adipose tissue-derived stem cells (ASCs) are multipotent mesenchymal stem cells with potent immunosuppressive properties, and their use as novel cell-based therapeutics have been proposed in many immune diseases. However, the analgesic effect and efficacy of ASCs to treat neuropathic pain remain unclear. This study, thus, investigated whether ASCs or ASC-derived culture medium can relieve neuropathic pain behaviors (i.e., mechanical and cold allodynia) in a rat model with L5 spinal nerve ligation. Intrathecal injection of ASCs significantly reduced cold allodynia, but not mechanical allodynia. Importantly, cold allodynia was completely reversed in rats with repeated injections of ASCs. In contrast, intrathecal injection of ASC-derived culture medium or *retro*-orbital injection of ASCs had no effect on neuropathic pain behaviors. These results suggest a novel and alternative therapeutic application of ASCs to target specific neuropathic pain behaviors.

## 1. Introduction

Current therapies for neuropathic pain are inadequate, with low efficacy and detrimental side effects. Although most of these therapies target pain transduction and transmission in the neurons, it is now clear that neuropathic pain is a neuroimmune [1]. New immune-based therapies have been tested to treat neuropathic pain, including mesenchymal stem cells (MSCs) [2]. In particular, a recent study demonstrated that intrathecal bone marrow stromal cell-derived multipotent stem cells/bone marrow-derived stem cells produce powerful analgesic effects in animal models of inflammatory pain and neuropathic pain by modulating neuroinflammation [3].

Adipose tissue-derived stem cells (ASCs) are multipotent mesenchymal stem cells, similar to bone marrow-derived stem cells, and can differentiate into various cell types, including osteocytes, adipocytes, neural cells, vascular endothelial cells, cardiomyocytes, pancreatic cells, and hepatocytes [4]. The main advantage of ASCs is that they can be easily obtained from the adipose tissue, and because of their genetic stability, they can used in auto- or allotransplantation [5]. Importantly, ASCs exhibit neuroprotective and immunosuppressive properties by modulating neurotrophic factors and pro- or anti-inflammatory cytokines and by interacting with neuronal and immune cells [6]. Thus, ASCs are considered one of the most promising cell types for cell-based therapies in cases of tissue failure, immune diseases, and neuronal pathologies. However, the effect and efficacy of ASCs for relieving and treating neuropathic pain remain unclear.

Thus, in this study, we investigated the efficacy of ASCs or ASC-derived culture medium to relieve neuropathic pain caused by mechanical and cold allodynia in a rat model with L5 spinal nerve ligation. Both ASCs and ASC-derived culture medium were tested to assess whether the modulation of neuropathic pain behaviors was mediated by the cells themselves or cell secretions. We also tested the efficacy of *retro*-orbital and intrathecal injection of ASCs to alleviate neuropathic pain. As a result, intrathecal injection of ASCs significantly reduced cold allodynia, but not mechanical allodynia. Thus, ASCs have a novel and alternative therapeutic agent to target cold allodynia in neuropathic pain.

## 2. Materials and Methods

### 2.1. Reagents

We used Dulbecco's phosphate-buffered saline (DPBS; Gibco, USA), penicillin streptomycin (Sigma, USA), collagenase-IV (Sigma, USA), 0.25% trypsin/1 mM ethylenediaminetetraacetic acid (Gibco-BRL, USA), Dulbecco's modified Eagle's high-glucose medium (DMEM, 4.5 g/L glucose; Gibco-BRL, USA), and fetal bovine serum (FBS; Gibco-BRL, USA).

### 2.2. Animals and Surgery

All surgical and experimental procedures were reviewed and approved by the Institutional Animal Care and Use Committee of the College of Medicine at Gachon University. Sprague-Dawley (150 g) rats, aged 6 to 10 weeks, were obtained from Samtako (Osan-si, Korea). The rats were used for the behavior analysis and primary culture. All rats were raised in a room maintained at a 12-h light/dark cycle (lights on at 7 : 00 AM), with a temperature of 22 to 25°C, and had ad libitum access to food and water. The bedding, food, and filtered water were changed twice a week. L5 spinal nerve ligation was performed according to the procedure described by Kim and Chung et al. [7]. Under isoflurane anesthesia (2% for induction and 0.5% for maintenance), following the exposure of the dorsal vertebral column from L4 to S2, the L6 vertebral transverse process was excised, and the L5 spinal nerve was then tightly ligated using a 6-0 silk thread under a dissecting microscope.

### 2.3. Primary Culture of ASCs

Under sterile conditions, the adipose tissue from regions close to the ovarian infundibulum in females and from the lateral epididymis region in males was extracted, as was the dermo-adipose tissue from the regions below the dermis and epidermis. The tissue was mechanically macerated using microscissors in a 100 mm petri dish with DPBS (DPBS; Gibco, USA). The tissue was incubated with IV collagenase-IV (0.075 mg/ml) (Sigma, USA) for 45 min at 37°C in 5% CO_2_ incubator. Thereafter, the tissue was centrifuged at 4000 rpm at room temperature (RT) for 3 min, the debris eliminated, and the cell pellet suspended in 5 ml of complete culture medium and filtered through a 100 *μ*m membrane. The filtered cells were centrifuged at 2000 rpm at RT for 2 min, and the supernatant was aspirated and resuspended in 1 ml of complete medium. The cell pellet was suspended in 1 ml of complete culture medium and plated onto a 100 mm culture dish. ASCs were quantified by hemocytometry, and all experiments were conducted with ASCs that had undergone more than four passages and verified by surface marker staining by flow cytometry.

### 2.4. Identification of ASCs by Flow Cytometry

ASCs were harvested, washed with 0.01 M PBS, and had cell density adjusted to 1 − 5 × 106 cells/ml in ice-cold PBS, 10% fetal calf serum (FCS), and 1% sodium azide. Cells were incubated with 0.1-10 *μ*g/ml fluorescein isothiocyanate- (FITC-) conjugated monoclonal antibodies against CD105, CD54, CD44, and CD45 for at least 30 minutes at RT, and isotype-matched FITC-conjugated antibodies were used as controls [5]. The cells were washed thrice with PBS, centrifuged at 400 rpm for 5 minutes, and resuspended in 500 *μ*l to 1 ml of PBS, 10% FCS, and 1% sodium azide and kept on ice under dark conditions. The properties of ASCs were analyzed using FacsCalibur (Biosciences).

### 2.5. Administration

Before the administration of ASCs, cells were cultured in DMEM without FBS and penicillin-streptomycin for 3 days and, thereafter, washed thrice with PBS, centrifuged at 2,000 rpm for 5 minutes, and then resuspended in DMEM. While under anesthesia, the resuspended ASCs (1 × 10^6^ cells/30 ul) or reagents (DMEM; 20 *μ*l) were injected into the right *retro*-orbital sinus of the rat using a 31-gauge insulin syringe [8]. For intrathecal injections, a spinal cord puncture was performed with a 31-gauge needle between the L5 and L6 levels to deliver ASCs (1 × 10^6^ cells in 30 *μ*l DMEM), ASC-derived culture medium (30 *μ*l medium obtained from 1 × 10^6^ cells), or vehicle (DMEM; 30 *μ*l) to the cerebrospinal fluid (CSF). Successful injection was verified by the tail-flick response immediately after inserting the needle [9].

### 2.6. Behavioral Analysis

The animals (*n* = 5 − 7 rats/group) were habituated to the testing environment for at least 3 days before baseline testing. The room temperature and humidity remained stable for all experiments. All pain symptoms were tested blindly. To assess mechanical sensitivity, we confined the rats in experimental cages on an elevated metal mesh stand and stimulated the plantar surface of the left hind paw with a series of von Frey hairs (0.4-15 g, Stoelting) and then evaluated the 50% withdrawal threshold by Dixon's up-down method [10]. To test cold sensitivity, we assessed the pain response by spraying 100 *μ*l of acetone onto the plantar surface of the left hind paw, placed on the mesh stand. Acetone was sprayed twice on the hind paw. The response to acetone was graded based on the following 4-point scale: 0, no response; 1, quick paw withdrawal reflex; 2, prolonged paw withdrawal or repeated flicking; 3, paw licking [11].

### 2.7. Statistical Analyses

Data are presented as mean ± SEM. Statistical analysis was performed using the Graph prism, version 6.1 (GraphPad Software, Inc, San Diego, CA). Behavioral data were analyzed by 2-way, repeated-measures ANOVA, followed by Sidak's posthoc test. The threshold for statistical significance was a *p* value <0.05.

## 3. Results

### 3.1. Identification of ASCs

**Figure 1:**
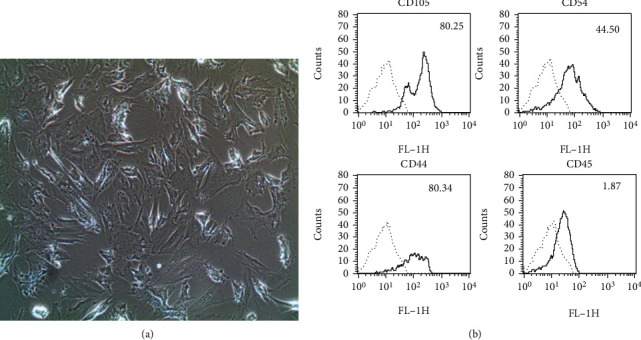
The characterization of rat ASCs. Representative image of rat ASC cultures at passage four, exhibiting a spindle-shaped, fibroblastic morphology. Magnification, ×200. (a) Flow cytometry analysis of cultured ASCs at passage four. Note that 80% of the cultured ASCs were positive for CD105 and CD44, except CD54, and only 1.87% of the cells were positive for CD45. (b) Isotype-matched FITC-conjugated antibody was used as the negative control for flow cytometry. ASC: adipose tissue-derived stem cell; FITC: fluorescein isothiocyanate.

Our ASC cultures were adult primary MSCs derived from rat subcutaneous visceral fat. To validate the origin of these cells, we used flow cytometry to assess the expression of cell surface lineage markers before conducting behavioral analysis. We validated the ASCs for four passages (see Figure 1(a)) by fluorescent immunostaining for CD105, CD54, CD44 (positive surface markers for mesenchymal cells or pluripotency), and CD45 (a positive marker for hematopoietic cells). Comparing with controls using isotype-matched FITC-conjugated antibodies, we found that the cultured ASCs were positive for CD105 (80.25%), CD44 (80.34%), and CD45 (44.50%), and that only 1.87% of the cells were positive for CD45 (see Figure 1(b)). Thus, we positively confirmed the identity of our cultured cells as ASCs.

### 3.2. Effect of ASCs on the Neuropathic Pain Behavior in a Rat Model with L5 Spinal Nerve Ligation

We induced neuropathic pain in rats by ligating the L5 spinal nerve [7]. The L5 spinal nerve ligation model is considered as one of the most suitable models for analgesic screening for neuropathic pain. Furthermore, mechanical or cold allodynia characterized neuropathic pain in this model. Moreover, we performed *retro*-orbital and intrathecal injections for the systemic and local ASC administration, respectively, in rats with the ligated L5 spinal nerve in order to identify the optimal delivery route conferring maximum analgesic efficacy of ASCs.

#### 3.2.1. *Retro*-Orbital Injection of ASCs Had No Effect on Pain Behavior

**Figure 2:**
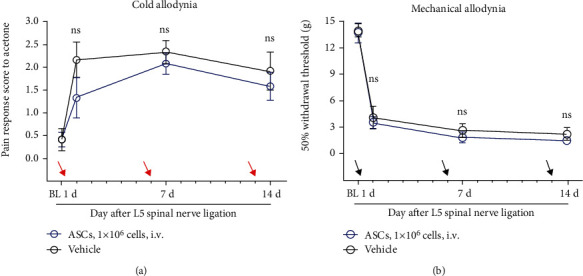
Absence of analgesic effect following the *retro*-orbital injection of ASCs on the rat model with L5 spinal nerve ligation. (a, b) No effect observed on cold allodynia (a) and mechanical allodynia (b) by treatment with ASCs (1 × 10^6^ cells) after the L5 spinal nerve ligation surgery. Arrows indicate time of injection (POD 0, POD 6, POD 13). *p* > 0.05, compared with vehicle (20 *μ*l DMEM); *p* > 0.05, compared with *n* = 6 rats/group. All data are expressed as the mean ± SEM. Statistical significance was determined by 2-way ANOVA, followed by Sidak's posthoc test. ns: not significant; POD: postoperative day; BL: baseline; i.v. : intravenous injection; ASC: adipose tissue-derived stem cell; DMEM: Dulbecco's modified Eagle's high-glucose medium.

To confirm the attenuation of the pain behavior after the administration of ASCs, ASCs (1 × 10^6^ cells in 20 *μ*L DMEM; *n* = 6 rats) or vehicle (20 *μ*L DMEM; *n* = 6 rats) was injected into a blood vessel via the right *retro*-orbital sinus on postoperative days (PODs) 0, 6, and 13. We confirmed that the *retro*-orbital injection of ASCs did not decrease cold or mechanical allodynia, despite repeated administration (see Figure 2(a) [interaction: *F*_(3, 15)_ = 1.276, *p* = 0.3185; group: *F*_(1, 5)_ = 3.055, *p* = 0.1409, 2-way ANOVA followed by Sidak's posthoc test; time: *F*_(3, 15)_ = 9.711, *p* = 0.0008]; Figure 2(b) [interaction: *F*_(3, 15)_ = 0.04517, *p* = 0.9867; group: *F*_(1, 5)_ = 1.032, *p* = 0.3563; time: *F*_(3, 15)_ = 125.6, *p* < 0.0001]; 2-way ANOVA followed by Sidak's posthoc test).

#### 3.2.2. Intrathecal Injection of ASCs Significantly Reduced Acetone-Induced Cold Allodynia

**Figure 3:**
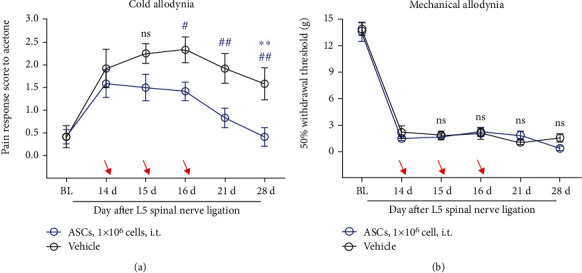
Intrathecal injection of ASCs specifically reduced cold allodynia in L5 spinal nerve ligation models. (a, b) Intrathecal injection of ASCs (1 × 10^6^ cells in 30 *μ*l DMEM) had an analgesic effect on cold allodynia (a) but not mechanical allodynia (b), administered 14 days after L5 spinal nerve ligation. ^#^*p* < 0.05, ^##^*p* < 0.01, ^###^*p* < 0.001, compared with vehicle (DMEM; 30 *μ*l); ^∗^*p* < 0.05, compared with POD14; *n* = 6 rats/group. Arrows indicate time of injection (POD 14, POD 15, POD 16). All data are expressed as the mean ± SEM. Statistical significance was determined by 2-way ANOVA, followed by Sidak's posthoc test. POD: postoperative day; BL: baseline; i.t. : intrathecal injection; ASC: adipose tissue-derived stem cell; DMEM: Dulbecco's modified Eagle's high-glucose medium.

Since the *retro*-orbital injection of ASCs did not inhibit neuropathic pain, we considered its inability to cross the blood-brain barrier as a possible reason for this. Therefore, we selected an intrathecal mode of the ASC administration to test the analgesic efficacy of ASCs because intrathecal injections directly deliver the injectate to the injured nerve. We administered ASCs (1 × 10^6^ cells in 30 *μ*L DMEM; *n* = 6 rats) into the cerebrospinal fluid by three repeated intrathecal injections and, subsequently, conducted neuropathic pain behavioral tests. We verified that, when compared with the vehicle group (30 *μ*L DMEM; *n* = 6 rats), the intrathecal administration of ASCs significantly reduced the scores of acetone-induced cold allodynia but not mechanical allodynia (see Figure 3(a) [interaction: *F*_(4, 20)_ = 1.425, *p* = 0.2622; time: *F*_(4, 20)_ = 3.411, *p* = 0.0279; group: *F*_(1, 5)_ = 14.90, *p* = 0.0119]; Figure 3(b) [interaction: *F*_(4, 20)_ = 2.811, *p* = 0.0531; time: *F*_(4, 20)_ = 2.694, *p* = 0.0604; group: *F*_(1, 5)_ = 0.3049, *p* = 0.6046]; 2-way ANOVA by Sidak's posthoc test). Thus, we concluded that intrathecal injection of ASCs has an analgesic effect on cold allodynia in the rat model of neuropathic pain.

#### 3.2.3. ASC-Derived Culture Medium Had No Effect on Pain Behavior

**Figure 4:**
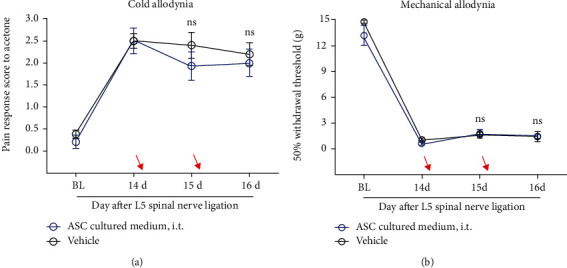
Absence of the analgesic effect following the injection of ASC-derived culture medium on the rat model with L5 spinal nerve ligation. (a, b) No effects observed on mechanical allodynia (a) and cold allodynia (b) by treatment with ASC-derived culture medium after the L5 spinal nerve ligation surgery. Arrows indicate time of injection (POD 14, POD 15). *p* > 0.05, compared with vehicle (30 *μ*l DMEM); *n* = 5 − 7 rats/group. *p* > 0.05, compared with POD14. All data are expressed as the mean ± SEM. Statistical significance was determined by 2-way ANOVA, followed by Sidak's posthoc test. ns: not significant; POD: postoperative day; BL: baseline; i.t.: intrathecal injection; ASC: adipose tissue-derived stem cell; DMEM: Dulbecco's modified Eagle's high-glucose medium.

ASCs have been reported to have regenerative and immunosuppressive effects, mediated by the release of anti-inflammatory cytokines, such as IL-10, in pathological conditions like neurodegenerative diseases [6]. To confirm the alleviation of cold allodynia by ASC-derived culture medium in our neuropathic pain model, we cultured ASCs (1 × 10^6^ cells) in serum-free medium for 3 days. Thereafter, we collected the cell-free culture medium and subsequently performed pain behavioral tests in experimental rats after intrathecal injection of this culture medium. We observed that the intrathecal injection of this ASC culture medium (30ul; *n* = 7 rats) showed no effect on cold allodynia in the neuropathic pain model, compared with vehicle (30ul DMEM; *n* = 5 rats), contrary to what was observed with the intrathecal administration of ASCs (see Figure 4(a) [interaction: *F*_(2, 20)_ = 0.6652, *p* = 0.5252; time: *F*_(2, 20)_ = 2.193, *p* = 0.1377; group: *F*_(1, 10)_ = 0.4253, *p* = 0.5290]; Figure 4(b) [interaction: *F*_(2, 20)_ = 0.5780, *p* = 0.5701; time: *F*_(2, 20)_ = 5.312, *p* = 0.0141; group: *F*_(1, 10)_ = 0.03669, *p* = 0.8519]; 2-way ANOVA followed by sidak's posthoc test). Thus, we concluded that ASCs directly and selectively induce analgesic effects to alleviate neuropathic pain.

## 4. Discussion

Current therapies have poor efficacy for neuropathic pain. Herein, we suggest that intrathecal injection of ASCs could be a novel and alternative cell-based treatment for neuropathic pain.

A recent study reported that intravenous injection of human ASCs inhibited pain-related behavior in a chronic constriction injury (CCI) mouse, STZ-diabetic mouse, and rat model of spinal cord injury for neuropathic pain [12–14]. Also, other clinical studies reported that intraarticular injection of human adipose-derived stem cells reduced pain responses without serious side effects [15, 16]. Although several animal studies demonstrated the efficacy and safety of hASC in chronic pain, the analgesic effects of cultured rodent primary ASCs were not fully evaluated. Our findings demonstrated that repeated intrathecal transplantation of cultured primary rat ASCs selectively attenuated cold allodynia, although *retro*-orbital injection of ASCs and intrathecal injection of ASC-derived culture medium failed to attenuate cold and mechanical allodynia in the L5 spinal nerve ligation model of neuropathic pain in rats.

Cold allodynia, i.e., an increased sensitivity to innocuous cold stimulus, is a characteristic feature of neuropathic pain. The transient receptor potential (TRP) channel family of proteins has been proposed to play an important role in mediating thermal-sensation in mammals. The transient receptor potential melastatin 8 (TRPM8) is a cold-sensor and ligand-gated cation channel [17]. Recent results of TRPM8-mediated analgesia are supported by data from cold pain tests performed on the chronic pain model of TRPM8 null mice or TRPM8 silenced rats [18, 19]. Although it is unknown if ASCs can target TRP channels, including TRPM8, our results suggest that autotransplantation of ASCs may alleviate cold allodynia in rats with L5 spinal nerve ligation, possibly by TRPM8 pathway inhibition.

Unlike MSCs, the greatest advantage of ASCs is that they can be easily obtained by less invasive procedures. ASCs are located in the subcutaneous adipose tissue, can be acquired by liposuction [20], and a significant number of ASCs can be obtained. Despite these advantages, there are many obstacles that need to be overcome for successful clinical translation. Primarily, in view of the significant analgesia provided by intrathecal injection of ASCs in rat models, the spinal implantation method may be preferred over other routes of injection. A previous study reported that direct intraspinal implantation of forebrain GABAergic neural progenitor cells attenuated neuropathic pain by fusing with the pain circuits of the spinal cord [21]. Although favorable analgesia was observed in this study, intraspinal injection of stem cells may be more appropriate for specific cases of spinal cord injury. There are several advantages of intrathecal administration of ASCs for managing peripheral neuropathic pain. First, it is minimally invasive in comparison with direct injection of ASCs into the injured spinal cord. Second, the small intrathecal space allows only for injection of limited amounts of injectate to reach therapeutic concentrations. Third, the intrathecal space is protected by the blood-spinal cord barrier and allows minimal infiltration of immune cells. Therefore, intrathecal injection of ASCs is one of the most important factors for managing neuropathic pain.

In contrast, our findings show that *retro*-orbital injection of ASCs did not confer analgesia in rats with L5 spinal nerve ligation. These results indicate that ASCs fail to cross the blood-brain barrier (BBB) and reach the site of nerve damage. Moreover, irrespective of the ability of the injected ASCs to cross the BBB, even the ASCs in the blood had no effect on the modulation of pain behavioral responses. Therefore, our findings suggest that *retro*-orbital injection of ASCs may be not the optimal delivery method for attenuation of neuropathic pain.

The implanted ASCs possess potent migratory abilities in pathological conditions. We confirmed that the injection of ASC-derived culture medium did not affect the pain-related behavior in L5 spinal nerve ligated rats with chronic pain, although we did not quantify the immuno- or neuronal modulators that may have been secreted into the culture medium due to serum starvation. This may be due to the direct action of ASCs on the injured tissue in cases of neuropathic pain. A recent study supported the hypothesis that ASCs express high levels of the chemokine receptor, C-X-C chemokine receptor type 4 (CXCR4), which binds stromal cell-derived factor-1*α* (SDF-1*α*), a secreted chemokine expressed by injured tissue. This mechanism might enable the migration of ASCs to the site of injury. Moreover, MSCs, similar to ASCs, have been demonstrated to migrate to the injured dorsal root ganglia via SDF-1*α*/CXCR4 signaling in a chronic pain model [3]. Implanted ASCs may differentiate to the neuronal cell-type of the injured location. A study reported that ASCs differentiate to neural cells, release neurotrophic factors such as BDNF, and induce nerve healing at the site of nerve injury [22]. Although further research is needed to support our findings, we speculate that the injected ASCs may migrate to the injured site and differentiate to neuronal cells and release specific neurotrophic factors for reducing TRPM8-mediated cold allodynia.

Recently, the convergence of the fields of nanoparticles and stem cells has shown significant possibilities for the study, diagnosis, and treatment of neurodegenerative disorders [23, 24]. Results of multiple studies support the notion that MSCs in combination with nanoparticle-mediated molecule delivery to the injured tissue may reduce unwanted side effects in that area [25]. Thus, it is critical to enhance ASC delivery to effectively reduce cold allodynia and other types of pain associated with neuropathy. Therefore, the use of nanoparticles may be considered to enhance the efficacy and delivery of ASCs.

## 5. Conclusions

In summary, these findings demonstrate the potent analgesic properties of intrathecal ASC transplantation for neuropathic pain, although efficacy was limited to cold allodynia. These cells can be further engineered to produce and secrete neuroprotective and inflammatory mediators, which could attenuate other types of allodynia, including cold allodynia. Moreover, enhancing ASC delivery may further improve the analgesic properties of implanted ASCs.

## Data Availability

The datasets used and/or analyzed during the current study are available from the corresponding author on reasonable request.

## Abbreviations

ASC: Adipose tissue-derived stem cell
CSF: Cerebrospinal fluid
DMEM: Dulbecco's modified Eagle's high-glucose medium
DPBS: Dulbecco's phosphate-buffered saline
FBS: Fetal bovine serum
TRP: Transient receptor potential
TRPM8: Transient receptor potential melastatin 8
SDF-1*α*: Stromal cell-derived factor-1*α*
CXCR4: C-X-C chemokine receptor type 4
BDNF: Brain-derived neurotrophic factor
POD: Postoperative day
i.t.: Intrathecal injection
i.v.: Intravenous injection.
